# New developments in the pathology of malignant lymphoma: a review of the literature published from June–August 2016

**DOI:** 10.1007/s12308-016-0284-5

**Published:** 2016-09-30

**Authors:** J. Han van Krieken

**Affiliations:** Department of Pathology, Radboud University Medical Centre, P.O. Box 9101, 6500 HB Nijmegen, The Netherlands

## Introduction

The yield of the literature this summer is a bit lower than normal, maybe due to a somewhat more critical selection of articles. Nevertheless, several interesting new data on recurrent lymphoma, the role of viruses, and the superiority of molecular features over clinical data might be helpful for the readership of the *Journal of Hematopathology*.

## Biology of lymphoma

### B cell lymphomas

Genomic instability is very common in cancer, including lymphomas, although the results of instability are rather stable. The control of the mitotic spindle assembly is therefore a very relevant process and a complicated one. This process is not only affected in many cancers but also the target of therapy. Engel et al. [1] demonstrate that USP9X is the mitotic deubiquitinase of the X-linked inhibitor of apoptosis protein (XIAP) and that deubiquitylation and stabilization of XIAP by USP9X lead to increased resistance toward mitotic spindle poisons. Furthermore, primary human aggressive B cell lymphomas exhibit high USP9X expression, which correlates with XIAP overexpression and high USP9X/XIAP expression is associated with shorter event-free survival in patients treated with spindle poison-containing chemotherapy. Finally, aggressive B cell lymphoma cell lines with USP9X and XIAP overexpression exhibit increased chemoresistance, reversed by specific inhibition of either USP9X or XIAP. Therefore, USP9X and XIAP are potential predictive biomarkers and targets in combined therapeutical approaches in aggressive B cell lymphoma.

Another approach to discover a potential predictive biomarker was used by Joosten et al. [2]. It was reported that only a subset of patients with B cell respond to histone deacetylase (HDAC) inhibition. Using an extensive molecular approach on 26 B cell lines, they found that the Src tyrosine kinase Gardner-Rasheed feline sarcoma viral (v-fgr) oncogene homolog (FGR) is associated with resistance to HDAC inhibition. As functional proof, CRISPR/Cas9-mediated FGR knockout in resistant cells restored sensitivity. In silico analysis of B cell lymphoma samples (*n* = 1200) showed a wide range of FGR expression indicating that FGR expression might help to stratify patients; however, clinical data were not available, nor results from treatment with HDAC inhibition.

Mantle cell lymphoma (MCL) is characterized by common recurrences after therapy. Wu et al. [3] collected primary samples and recurrences from 13 patients with MCL and used whole genome sequencing in order to indentify the underlying genetic basis for recurrent disease. They confirmed that MCL is genetically heterogeneous (like all cancers) and also that genetic alterations acquired in the relapse samples are very different between patients. Juscevicius et al. [4] performed a similar study in diffuse large B cell lymphoma (DLBCL), comparing also cases with and without a relapse (20 of each). In 3 of the patients the relapse appeared actually a new primary lymphoma. In bona fide relapses, they found 2 distinct genetic routes: [1] early-divergent/branching evolution from a common progenitor in 6 patients and [2] late-divergent/linear progression of relapses in 11 patients. Analysis of recurrent genetic events identified potential early drivers of lymphomagenesis (KMT2D, MYD88, CD79B, and PIM1). The most frequent relapse-specific events were additional mutations in KMT2D and alterations of MEF2B. SOCS1 mutations were exclusive to non-relapsing DLBCL, whereas primaries of relapsing DLBCL more commonly displayed gains of 10p15.3-p12.1 containing the potential oncogenes PRKCQ, GATA3, MLLT10, and ABI1. Melchart et al. [5] addressed the same question, but used a more limited, targeted approach with 108 genes enabling the use of formalin fixed paraffin embedded material of 28 patients. Mutations were present in 74 of the 104 genes tested. Primary tumor samples showed a median of 8 mutations (range 0–24) with the used gene set. Lower numbers of mutations in the primary tumor were associated with a better median overall survival. They describe three patterns of clonal evolution toward relapse of disease: large global change, subclonal selection, and no or minimal change possibly suggesting preprogrammed resistance. Although, a new primary was not excluded, these results are in line with those of Juscevicius et al. [4], pattern 1 being comparable with route 2 and patterns 2 and 3 comparable with route 1; because of the fewer genes analyzed, indeed pattern 3 may actually be a subtle variant of pattern 2.

Lymphomas originating in the thymus, derived from B cells, form an enigmatic group of entities. B cells are few in number in the normal thymus and are considered to be the cell of origin of mediastinal (m)DLBCL, which is confirmed with more detail by Bergkvist et al. [6]. They investigated the B cells from the bone marrow and thymus of non-lymphoma patients, obtained during cardiac surgery. In the thymus, 4 % (median; range 2–14 %) of the CD45+ cells were CD19+ B cells, with a major fraction being CD27+/CD38− memory B cells; for the bone marrow, this was 14 % (median; range 3–27 %), and only a minor fraction of memory B cells. Global gene expression analysis of the memory B cell subsets from the two compartments identified 133 genes higher in thymus, including AICDA, REL, STAT1, TNF family, SLAMF1, CD80, and CD86. This thymic memory B cell gene profile was also more often expressed in primary mDLBCL compared with other types. It would be interesting to know how these results relate to mediastinal Hodgkin lymphoma (HL) and the intermediate type (“grey zone”).

### T cell lymphoma

Margolsky et al. [7] performed targeted sequencing of 465 cancer-related genes and high-resolution copy number analysis in 19 post-transplant lymphoproliferative disorders (PTLD) of T or NK cell rare entities and their genetic basis is unclear. They detected 377 variants detected, with an average of 20 variants per case. The most frequent alterations were mutations of epigenetic modifier genes (TET2, KMT2C, KMT2D, DNMT3A, ARID1B, ARID2, KDM6B), inactivation of TP53 by mutation and/or deletion and mutations of JAK/STAT pathway genes (*n* = 5). Complex copy number changes were detected in 8 of 16 (50 %) cases and disease subtype-specific aberrations were also identified. In contrast to B cell PTLDs, the molecular and genomic alterations observed in T/NK-PTLD appear similar to those reported for peripheral T cell lymphomas (PTCL) occurring in immunocompetent hosts, which, according to the authors, may suggest common genetic mechanisms of lymphoma development. However, the number of cases is quite low. Boddicker et al. [8] investigated 148 PTCL with DNA and RNA next-generation sequencing to identify chromosomal rearrangements encoding expressed fusion transcripts in PTCL. Two of 11 cases had novel fusions involving VAV1, encoding a truncated form of the VAV1 guanine nucleotide exchange factor important in T cell receptor signaling. Fluorescence in situ hybridization (FISH) studies identified VAV1 rearrangements in 10 of 148 PTCLs (7 %). These were observed exclusively in PTCL, not otherwise specified (11 %) and anaplastic large cell lymphoma (11 %). They also identified novel kinase gene fusions, ITK-FER and IKZF2-ERBB4, as candidate therapeutic targets that show similarities to known recurrent oncogenic ITK-SYK fusions and ERBB4 transcript variants in PTCLs, respectively. Additional novel and potentially clinically relevant fusions also were discovered. The authors conclude that their identification of VAV1 fusions as recurrent and targetable events in PTCLs indicates the potential for their sequencing approach to guide individualized therapy approaches for this group of aggressive malignancies.

## Viruses and lymphoma

The role of several viruses in the pathogenesis of lymphomas is well known. The presence of a virus even defines some lymphoma types like Epstein-Barr virus (EBV)-positive DLBCL of the elderly. However, Monabati et al. [9] add further evidence that the age criterium is not relevant and that “elderly” needs to be omitted (see also [10, 11)]. Out of 95 their DLBCL, 11.6 % were EBV positive (7.5 and 14.5 % in the young and old groups). They did not find any significant difference in IHC subclasses and clinical data between EBV-positive DLBCL of young and old groups. Kadry et al. [12] compared 100 newly diagnosed Egyptian lymphoma patients with 100 healthy age- and sex-matched normal controls for the presence of hepatitis B and C (HBV, HCV) viruses, EBV, cytomegalovirus (CMV), and human herpes virus-8 (HHV-8). There was high indication of presence of most viruses in lymphoma cases except for positive HBs Ag, positive CMV IgG, and HHV-8. Surprisingly, no significant difference was found between Hodgkin (HL) and non-Hodgkin (NHL) patients except for HCV antigen, 57 % for HL and 27 % for NHL. Another virus that is related to lymphomas is the human herpes 6 virus (HHV-6), which can remain latent and chronic in the host cells after primary infection. Kiani et al. [13] found, using a nested PCR-method, 12/22 (54 %) cases of HL and 8/22 (36 %) NHL to be positive for HHV-6. What this exactly means with respect to pathogenesis and treatment remains to be studied.

Plasmablastic lymphoma (PL) is a rare and aggressive DLBCL commonly associated with EBV-infection that most often occurs in the context of human immunodeficiency virus infection. Laurent et al. [14] found that of their 82 PL cases half were EBV positive and that these had more often a MYC-rearrangement and a better 2-year event-free survival. Both the microenvironment (65 %) and the tumor cells (22 and 5 %) showed expression of programmed cell death-1 (PD-1) and programmed cell death-ligand 1 (PDL-1) with the EBV-positive cases more often positive. Whether the expression is related to response to (immune)therapy remains to be studied.

## Defining entities

### B cell lymphomas

In a series of papers, van den Brand et al. [15–18] draw attention to the relatively poorly characterized nodal marginal zone lymphoma (NMZL). Now, they analyze the role of nuclear factor kappa B (NF-kappaB) in 20 nodal marginal zone lymphomas (NMZLs), 20 follicular lymphomas (FLs), and 11 cases of low-grade B cell lymphoma, unclassifiable (BCL-u; [19)]. Their NMZLs were diagnosed with strict criteria including expression of at least one putative marginal zone marker (MNDA and/or IRTA1; [17]). Cases that showed features of NMZL but did not fulfill all criteria were included as BCL-u. All FLs were required to have a BCL2 rearrangement. NF-kappaB-pathway gene mutations were found in 9 NMZLs, with recurrent mutations in TNFAIP3 and CD79B. In FL, mutations were found in 12 cases, with recurrent mutations in TNFRSF14, TNFAIP3, and CARD11. In BCL-u, mutations were found in 5 cases with recurrent mutations in TNFRSF14. TNFRSF14 mutations were present in FL and BCL-u, but not in any of the NMZLs. In the group of BCL-u, TNFRSF14 mutations clustered with a FL immunophenotype. These results suggest that TNFRSF14 mutations point toward a diagnosis of FL and can be used in the sometimes difficult distinction between NMZL and FL, but, of course, to apply this in diagnostics would require confirmation in an independent cohort.

Primary mediastinal B cell lymphoma (PMBL) is an entity of B cell lymphoma distinct from the other molecular subtypes of diffuse large B cell lymphoma (DLBCL). However, without knowledge of the primary site of presentation this entity remains difficult to diagnose. Jardin et al. [20] investigated the prevalence, specificity, and clinical relevance of mutations of XPO1, which encodes a member of the karyopherin-β nuclear transporters. XPO1 mutations were present in 28/117 (24 %) PMBL cases and in 5/19 (26 %) HL cases but absent/rare in MZL (0/20) or DLBCL (3/197). A higher prevalence (50 %) of the recurrent codon 571 variant (p. E571K) was associated with shorter PFS, independent from age, International Prognostic Index, and bulky mass. The authors conclude that the XPO1 E571K mutation represents a genetic hallmark of the PMBL subtype of DLBCL.

Thymocyte selection-associated high-mobility group box (TOX) is aberrantly expressed in cutaneous T cell lymphomas. To evaluate whether TOX is also expressed by cutaneous B cell lymphomas, Schrader et al. [21] performed TOX immunohistochemistry on skin biopsies of 44 patients with primary and secondary cutaneous B cell proliferations. TOX was expressed not only in the reactive follicle center cells of lymph nodes, tonsils, cutaneous lymphoid hyperplasia, and primary cutaneous MZL but also by the neoplastic cells of 16/17 patients with primary cutaneous follicle center lymphoma (PCFCL) and 7/7 patients with cutaneous manifestations of systemic FL. Notably, TOX showed a very similar expression pattern as BCL6. In 4/10 patients with a BCL6(+) primary cutaneous diffuse large B cell lymphoma, leg type (PCDLBCL,LT) and in 2/2 patients with a secondary cutaneous BCL6(+) diffuse large B cell lymphoma (DLBCL), TOX was expressed by more than 50 % of the neoplastic B cells. In contrast, in 3/3 BCL6(−) PCDLBCL,LT, TOX was completely negative or weakly expressed by a minor proportion of the neoplastic B cells. In conclusion, TOX is expressed not only by neoplastic T cells but also by both reactive and neoplastic follicle center (germinal center) B cells and a proportion of BCL6(+) PCDLBCL,LT and secondary cutaneous BCL6(+) DLBCL. The authors concluded that the functional significance of TOX expression in reactive and neoplastic B cells remains to be elucidated. The marker is not very helpful in differential diagnosis.

## New entities/subtypes

IgG4-related disease (IgG4-RD) is mentioned in quite a few of these literature reviews and is now a recognized fibro-inflammatory disorder, which may affect many organs, and often comes to clinical attention due to tumor-like organ swelling. Typical histopathology of IgG4-RD is lymphoplasmacytic infiltration rich in IgG4-positive plasma cells (PCs), storiform fibrosis, and obliterative phlebitis. Patients with sicca symptoms can be misdiagnosed as primary Sjögren’s syndrome (pSS) instead of IgG4-RD because of clinical and histopathological similarities. Moreover, an association with lymphoma development is described in both diseases. Vasaitis et al. [22] investigated signs of IgG4-RD in a population-based cohort of patients diagnosed with pSS complicated by lymphoma from the Swedish Patient Register 1964–2007 and the Cancer Register 1990–2007 (*n* = 79). All lymphoma tissues and available minor salivary gland biopsies (*n* = 11) were immunostained for IgG4 and evaluated for other histopathological signs of IgG4-RD. Only one patient of 79 (1.3 %) had >10 IgG4 plasma PCs/high-power field (HPF) in the lymphoma tissue, and an unspecified low-grade B cell lymphoma localized in the submandibular gland. This patient had also other histopathological features of IgG4-RD in the lymphoma and a surgical lung biopsy taken 5 years before lymphoma diagnosis and, therefore, fulfilled the criteria for IgG4-RD. Occasional IgG4 positive PCs (<10/HPF) without signs of IgG4-RD were observed in another six lymphomas. No IgG4-positive PCs were identified in the minor salivary gland biopsies. Thus, histopathological findings of IgG4-RD may rarely co-exist with low-grade malignant B cell lymphoma in patients with initially suspected pSS and may occasionally be associated with an underlying IgG4-RD.

Pediatric-type nodal (PTN) FL is a variant of FL characterized by limited-stage presentation and invariably benign behavior despite often high-grade histological appearance. It is important to distinguish PTNFL from typical FL in order to avoid unnecessary treatment; however, this distinction relies solely on clinical and pathological criteria, which may be variably applied. Louissaint et al. [23] found, using copy number analysis and exome and/or targeted sequencing of 26 PTNFLs (16 pediatric and 10 adult), that the most commonly mutated gene in PTNFL was MAP2K1, encoding MEK1, with a mutation frequency of 43 %. All MAP2K1 mutations were activating missense mutations localized to exons 2 and 3, which encode negative regulatory and catalytic domains, respectively. Missense mutations in MAPK1 (2/22) and RRAS (1/22) were identified in cases that lacked MAP2K1 mutations. The second most commonly mutated gene in PTNFL was TNFRSF14, with a mutation frequency of 29 %, similar to that seen in limited-stage typical FL. PTNFL was otherwise genomically bland and specifically lacked recurrent mutations in epigenetic modifiers. Copy number aberrations affected a mean of only 0.5 % of PTNFL genomes, compared with 10 % of limited-stage typical FL genomes. Importantly, the mutational profiles of PTNFLs in children and adults were highly similar. Together, these findings define PTNFL as a biologically and clinically distinct indolent lymphoma of children and adults characterized by a high prevalence of MAPK pathway mutations and a near absence of mutations in epigenetic modifiers. Therefore, like the removal of elderly from EBV-positive lymphoma, mediastinal from those DLBCL, it seems pediatric needs to be removed from the PTNFL.

## Pitfalls in lymphoma diagnosis

Most B cell lymphomas express the B cell markers CD20, CD79a, and Pax 5, but occasionally one, two, or even all three markers are absent while there is genotypically a B cell neoplasia, especially anaplastic lymphoma kinase-positive DLBCL, PL, primary effusion lymphoma, and extracavitary human herpes virus 8 (HHV8)-positive DLBCL. Yin et al. [24] selected 34 cases of previously diagnosed B cell lymphomas with no or weak expression of CD20, CD79a, and PAX5 and found Oct2 and Bob1 to be positive in 74 % (25 of 34) and 85 % (29 of 34) of the cases, respectively. Ninety-four percent (32 of 34) of the cases expressed at least one of these two markers. None of the 51 control cases of non-B cell neoplasms expressed either Oct2 or Bob1. These data confirm that Oct2 and Bob1 may be helpful in defining rare cases of B cell neoplasia.

Nodal follicular helper T cell-derived lymphoproliferations (specifically the less common peripheral T cell lymphomas of follicular type) exhibit a spectrum of histologic features that may mimic reactive hyperplasia or HL. Alikhan et al. [25] found that nine of their 10 (90 %) peripheral T cell lymphomas of follicular type showed a CD3−/dimCD4+ T cell population constituting 29 % (range 7.9–62 %) of all lymphocytes. Five of 10 (50 %) had nodular lymphocyte predominant (LP)HL or lymphocyte-rich classical (c)HL-like morphology with scattered Hodgkin-like cells that expressed CD20, CD30, CD15, and MUM1. Three cases had a nodular growth pattern and three others exhibited a perifollicular growth pattern without Hodgkin-like cells. EBV was positive in 1 of the 10 cases (10 %). PCR analysis showed clonal T cell receptor gamma gene rearrangement in all 10 cases. In comparison, 11 of 15 (73 %) angioimmunoblastic T cell lymphomas showed the CD3−/dimCD4+ population (mean 20 %, range 3–72 %). Using a threshold of 3 % for CD3−/dimCD4+ T cells, all 15 LPHL and 8 cHL were negative, as were 25 of 26 reactive lymph nodes. The authors conclude that the high frequency of CD3−/dimCD4+ aberrant T cells is similar in angioimmunoblastic T cell lymphomas and peripheral T cell lymphomas of follicular type and is a useful feature in distinguishing peripheral T cell lymphomas of follicular type from morphologic mimics such as reactive hyperplasia or Hodgkin lymphoma.

## Prognostic factors in lymphoma

Kim et al. [26] provide data that may turn the molecular classification of DLBCL into ABC and GBC type from a pure prognosticator into a predictive marker. They used retrospective data from 219 newly diagnosed high-risk DLBCL patients, of whom 81 had received an autologous stem cell transplantation (ASCT) and 138 patients did not. As expected, the ASCT group had a better survival and patients with the ABC subtype had an inferior PFS than those with the GCB subtype. In the non-ASCT group, the ABC subtype showed also a worse survival, but in the ASCT group, there was no difference in survival according to molecular classification. This result suggests that upfront ASCT may improve the poor prognosis of non-GCB subtype in high-risk DLBCL.

Wong et al. [27] try to improve the molecular classification of DLBCL by showing that FOXP2-positive DLBCL indicates poor response to R-CHOP therapy, especially in the ABC subtype. Although their in vitro work gives a good biological explanation, it will be difficult to introduce this marker into practice.

Green et al. [28] have an even larger ambition: they want to improve the IPI score by adding LMO2 and BCL2 transcription levels. Although their statistical approach is sound, it is even more unlikely that such an approach will enter the clinic.

Duncan et al. [29] take a more traditional approach by analyzing protein expression of Runt-related transcription factor-3 (RUNX3) and enhancer of zeste homolog-2 (EZH2), a histone methyltransferase, which has been shown to mediate silencing of RUNX3. They found in 83 DLBCL cases loss of RUNX3 in 20 cases; EZH2 expression was observed in 59 cases. RUNX3-negative tumors had a lower survival. They conclude that further studies are warranted to elucidate the biology and prognostic utility of RUNX3 in DLBCL, a conclusion that actually is not very promising.

## Ancillary techniques

Clonality testing can be very helpful, provided that it is used correctly. Roepman et al. [30] assessed the routine diagnostic value of the EuroClonality/BIOMED-2 assay for B cell clonality on 192 air-dried archived Giemsa-stained smears obtained by fine needle aspiration from 184 patients. The clonality assay showed a high accuracy of 93 % for detection of malignancy, with a sensitivity of 93 % and a specificity of 92 %. All 64 cases with monoclonal Ig heavy chain/Ig kappa chain rearrangements were confirmed to be malignant by histology or clinical follow-up. Expert re-evaluation of the gene scan-data changed the definite diagnosis for five cases (3 %), mainly because of low signals or absence of proper duplicate results. This study shows that EuroClonality/BIOMED-2 assay can successfully be performed on cytological Giemsa-stained smears and inclusion in daily practice can assist in better identification of malignant lymphoma, provided that sufficient expertise is available.

Liew et al. [31] evaluated the value of a digital FISH capture and imaging system for the detection of MYC 8q24 translocations using LSI-MYC (a break-apart probe) and MYC 8;14 translocation using IGH-MYC (a fusion probe). The LSI-MYC probe was tested on tissue sections from 35 patients and the IGH-MYC probe on 40 patients. Results for LSI-MYC had a high degree of correlation between traditional method of FISH analysis and digital FISH analysis. Results for IGH-MYC had a 100 % concordance between traditional method of FISH analysis and digital FISH analysis.
